# A Systematic Review of Self-Reported Outcome Measures Assessing Disability Following Hand and Upper Extremity Conditions in Persian Population

**DOI:** 10.22038/abjs.2020.48859.2423

**Published:** 2021-03

**Authors:** Erfan Shafiee, Maryam Farzad, Mahdieh Karbalaei

**Affiliations:** 1 School of Physical Therapy, Department of Health and Rehabilitation Sciences, University of Western Ontario, London, Ontario, Canada; 2 Roth McFarlane Hand and Upper Limb Centre, St. Joseph’s Hospital, University of Western Ontario, London, Ontario, Canada; 3 University of welfare and rehabilitation sciences, Tehran, Iran; 4 Department of Occupational Therapy, Tehran University of Medical Sciences, Tehran, Iran

**Keywords:** cross-cultural adaptation, Disability, hand and upper extremity, patient-reported outcome measure, psychometric properties

## Abstract

**Background:**

Disability following hand and upper extremity conditions is common. Patient-reported outcome measures (PROs) are used to capture patients’ status subjectively. This review has aimed to synthesis the literature regarding the extent and methodological quality of translation, cross-cultural adaptation, and psychometric properties of the hand and upper extremity disability PROs in the Persian language.

**Methods:**

Seven electronic databases (MEDLINE, EMBASE, Psychinfo, Scopus, ISI, Science direct, and Google Scholar) were searched until May 2020. Studies reporting cross-cultural adaptation and psychometric properties testing of the Persian validated disability PROs of the hand and upper extremity were identified. We appraised the eligible studies using Guidelines for the Process of Cross-cultural Adaptation of Self-report Measures and COnsensus-based Standards for the selection of health Measurement INstruments (COSMIN) risk of bias checklist.

**Results:**

Out of 98 identified records, 22 studies on 17 PROs were reviewed. Most of the PROs (47%) were region-specific and the others were condition-specific (29%) and multi-region (24%). Most of the studies (67%) followed 80 to 100% of the recommended steps for cross-cultural adaptation and translation of a PRO. The evidence of internal consistency, test-retest reliability, and construct validity was available for all the PROs. Structural validity, measurement error, and responsiveness were evaluated for five, six, and four PROs, respectively. The overall risk of bias ranged from “inadequate” to “very good” for all studies.

**Conclusion:**

A reasonable number of PROs for the evaluation of hand and upper extremity disability are available in the Persian language. Although all of them are not of very good psychometric properties, they all have sufficient quality to be used in clinical settings.

## Introduction

and and upper extremity conditions are common in general population and are often associated with disability and pain (1–3). Hand and upper extremity injuries have a more significant impact on disability and restrictions in performing routine activities compared with the other parts of the body (4, 5). Therefore, the focus of interventions in hand and upper extremity conditions are improving the function and decreasing the disability level (3). Patient-reported outcome measures (PROs) are widely used to measure the impact of hand surgery and hand therapy interventions from patients’ perspectives (6). 

Disability is a broad term that includes objective and subjective folds. The subjective dimension of disability could be evaluated with a valid PRO to capture patients’ subjective feelings of their current status (7). The PROs evaluating disability are supplemental materials to the objective clinical assessment and quantify patients’ perceived restrictions (8). 

Most of the common, valid, and reliable outcome measures for the evaluation of disability in hand and upper extremity conditions are published in English (9). Adapting available outcome measures with well-documented psychometric properties to different languages and cultures is more feasible than creating a new one (10). However, nonequivalent words, idiomatic expressions, and cultural backgrounds can cause problems in the process of translations. To overcome these problems, it is essential to have a clear distinction between translation and cross-cultural adaptations (11). The cross-cultural adaptation process should be done based on standard guidelines to achieve an equivalent translated outcome measure (10, 12). 

Testing the psychometric properties (reliability, validity, and responsiveness) of a cross-culturally adapted measure is needed to confirm the appropriateness of using that adapted measure in the target population (13). There are several adapted and validated PROs in the Persian population to evaluate disability and function in hand and upper extremity conditions. 

The aim of this review was to critically appraise, compare, and summarize the quality of the psychometric properties of Persian validated PROs assessing disability following hand and upper extremity conditions.

## Materials and Methods

A broad search strategy was performed to identify the outcome measures available in Persian for the evaluation of disability following hand and upper extremity conditions. Seven electronic databases were used: EMBASE, MEDLINE, Psychinfo, Scopus, ISI, Science direct, and Google Scholar from beginning to April 2020. Search keywords were as follows: “Cross-cultural adaptation OR Translat*” AND “Psychometric propert* OR Measurement” AND “Reliability OR Valid* OR Responsiveness OR Rasch analysis OR Factor analysis” AND “Persian OR Farsi OR Iran*” AND “Disability OR Function” AND “Hand OR Wrist OR Forearm OR elbow OR Arm OR Shoulder OR upper extremit* OR limb”. References and citations of the included papers and recent systematic reviews were checked for additional studies, and forward and backward citation tracing was used. This review was registered on the PROSPERO (CRD42020179934). 

***Eligibility Criteria***

**Figure 1:**
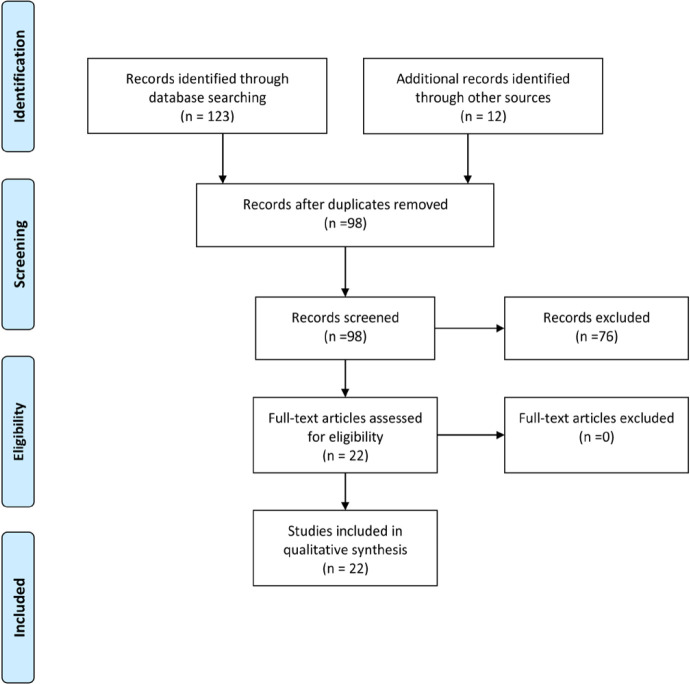
Flow diagram of the study selection

Studies focusing on cross-cultural adaptation process and/or measuring psychometric properties of the hand and upper extremity disability PROs were included. Studies that were not published in a peer-review journal or as a full manuscript were excluded. No limitation in the publication date or language was implemented. A flow diagram based on the Preferred Reporting Items for Systematic Reviews and Meta-analyses (PRISMA) presents the search strategies, and the number of studies included or excluded in the qualitative synthesis [Figure 1] (14).

***Data Extraction and Quality Assessment***

The process of data extraction was done by two independent reviewers (ES, MK) and checked by the third reviewer (MF). The reviewers investigated the eligible studies to extract demographic data of participants (age, male/female, type of injury), sample size, and population of the studies. The value of each psychometric property was also extracted. These data were presented to provide a description of the conditions that each study was conducted and also to evaluate the methodological quality of the studies. 

We extracted the data related to the psychometric properties of the PROs, including the structural validity, internal consistency, reliability, measurement error, criterion/construct validity, responsiveness, and floor or ceiling effects (15). 

The quality of the cross-cultural adaptation process was assessed using Guidelines for the Process of Cross-cultural Adaptation of Self-report Measures (10). The included studies were screened in terms of forward translation, synthesis, backward translation, expert committee review, and pilot testing (12). Each step was rated based on positive, negative, no information, or unclear rating scheme, which is defined elsewhere (16). 

In terms of evaluating the quality of measurement properties of the PROs, we used the COnsensus-based Standards for the selection of health Measurement INstruments (COSMIN) risk of bias checklist and criteria for good measurement properties (17). The COSMIN Risk of bias tool comprises ten checklists, including: (1) PROM development, (2) Content validity, (3) Structural validity, (4) Internal consistency, (5) Cross-cultural validity/Measurement invariance, (6) Reliability, (7) Measurement error, (8) Criterion validity, (9) Hypotheses testing for construct validity, and (10) Responsiveness. Regarding the aim of this review, we considered checklists 3 to 10 for the risk of bias assessment. Each checklist criterion was rated as “very good”, “adequate”, “doubtful”, “inadequate”, or “not applicable”. The overall rating of the quality of each PRO on a psychometric property was determined based on “the worst score counts” principle. Then we rated the quality of each measurement property against the COSMIN established criteria for good measurement properties as “sufficient”, “indeterminate”, and “insufficient”, which is defined in detail in the COSMIN manual (18).

Two independent reviewers (ES and MF) did the risk of bias and quality assessment. In case of disagreement, consensus was achieved through discussion with the third reviewer (MK).

## Results

Out of 98 identified records, 22 studies on 17 PROs were eligible for the aim of this review and data analysis. In total, 2672 participants in the shoulder (n=1508), elbow (n=425), and hand-wrist region (n=793) were included in the studies. In the multi-region PROs, 423 participants were included [Table 1].

Ten studies (45%, n=10/22) reported translation and psychometric properties of the seven PROs for the evaluation of disability in the shoulder: American shoulder and elbow surgeons (ASES) (19), Shoulder Pain and Disability Index (SPADI) (20),(21), Oxford shoulder score (OSS) (22), (23), Oxford Shoulder Instability (OSIS) (24), Simple shoulder test (SST) (23),(25), Western Ontario Rotator Cuff Index (WORC) (26), and Shoulder Activity Scale (SAS) (27). The WORC (26) and OSIS (24) are condition-specific PROs that evaluate disability in rotator cuff and shoulder instability conditions, respectively. 

Five studies (22%, n=5/22) reported the translation and psychometric properties of the three elbow disability PROs: Oxford Elbow Score (OES), Patient-Rated Elbow Evaluation (PREE), and Patient-Rated Tennis Elbow Evaluation (PRTEE) (28–32). The PRTEE is a condition-specific PRO for evaluating pain and function in tennis elbow.

Translation and psychometric properties of the five (22%, n=5/22) hand and wrist disability PROs were reported in six studies: Patient-Rated Wrist Evaluation (PRWE), Boston Carpal Tunnel Syndrome Questionnaire (BCTQ), Functional Index of Hand Osteoarthritis (FIHO), Patient-Rated Wrist/Hand Evaluation (PRWHE), and Michigan Hand Questionnaire (MHQ) (33-38). The BCTQ and FIHO are condition-specific PROs for carpal tunnel syndrome and osteoarthritis, respectively (35, 36).

Three studies measured psychometric properties of Disabilities of the Arm, Shoulder and Hand (DASH) and Quick-DASH as a multiregional PRO (20, 39, 40). One of the studies specifically evaluated the responsiveness of the DASH in the patients with shoulder problems [Table 2] (20). 

***Translation and cross-cultural adaptation***

Twenty-one studies reported translation and cross-cultural adaptation of the 17 disability PROs to the Persian language [Table 3]. The guidelines developed by Beaton et al. was followed in 15 studies (75%), two studies did not mention the method they had used (10, 19, 35). Wild et al. and World Health Organization methods for translation and cross-cultural adaptation were used in two studies (28, 37).

From 21 included studies, 100% followed the guidelines in forward translation. Nineteen (90%) studies were rated as positive in terms of synthesis, and the information about synthesis in two studies was unclear (31, 34). Backward translation in 9 studies (43%) were in accordance with the guidelines (23, 24, 26, 29, 31–33, 36, 39). To achieve cross-cultural equivalency, 16 studies (76%) synthesized the translations with the expert committee (19, 21–24, 26, 28–34, 37–39). Testing the pre-final version of the PROs was done in 17 (80%) studies (21, 23–29, 31–39).

Psychometric properties testing - Quality and risk of bias assessment: 

***Structural validity***

Four studies evaluated the structural validity of five PROs (The DASH, OSS, SST, SAS, and MHQ) by either confirmatory factor analysis (CFA) or exploratory factor analysis (EFA) (23, 27, 38, 39). None of the studies used Rasch analysis to test the dimension or factor structure of the PROs. Regarding the risk of bias and quality assessment, the structural validity was rated adequate and indeterminate for four PROs (the DASH, OSS, SST, and MHQ), respectively [Table 4] (23, 38, 39). 

***Internal consistency***

20 Out of 22 studies (90%) reported Cronbach’s alpha as the index of internal consistency for all PROs. Cronbach’s alpha for the DASH and Q-DASH ranged from 0.90 to 0.96. 

In the shoulder disability PROs, Cronbach’s alpha was reported in eight studies ranging from 0.64 to 0.94 (19, 21–27, 39, 40). The lowest score was reported for the SAS (27) (Cronbach’s alpha=0.64), and the highest score was reported for the SPADI ( Cronbach’s alpha=0.94).

The values of Cronbach’s alphas in the elbow disability PROs ranged from 0.91 to 0.98. In the hand and wrist disability PROs the range of Cronbach’s alpha was from 0.79 to 0.93 (21, 27, 29–32, 34–38). 

Regarding the risk of bias assessment, 15 studies (75%) were rated as very good (21–30, 35, 36, 38–40). The value of Cronbach’s alpha in 14 studies (70%) was calculated for each unidimensional scale or subscale (21–26, 28–30, 35, 36, 38–40). The quality of these 14 studies regarding internal consistency was rated as sufficient. Those that were rated as inadequate, had not calculated Cronbach’s alpha for each unidimensional subscale (ASES: two subscales, and PRWHE: two subscales) (19, 37). As no information was available on the structural validity of the PRTEE questionnaire and none of the Persian PRTEE versions conducted factor/Rasch analysis, the risk of bias assessment for internal consistency of the PRTEE was doubtful in all three studies (30–32).

***Reliability***

The Intraclass Correlation Coefficient (ICC) was reported in 90% (n=20 out of 22) of the included studies for all PROs. The ICC value ranged from 0.54 to 0.99. Assessing risk of bias in terms of reliability, two studies (10%) were rated as very good as they had provided evidence that patients were stable during the test-retest period (the SAS and FIHO) (27, 36). The other studies were rated as adequate and doubtful due to the lack of clear information about the time of conducting retest or status of patients at the time of retest. The value of ICC in two studies, was less than 0.7 (The SST and BCTQ symptom severity scale); therefore, they were rated as insufficient in terms of quality assessment (25, 35).

***Measurement error***

The values of Standard Error of Measurement (SEM), Minimal Detectable Change (MDC), and Minimal Clinically Important Difference (MCID) were reported in 7, 4, and 2 studies, respectively (19, 20, 23, 27, 30–32, 36, 37). The risk of bias in four studies was rated as adequate due to the lack of clear information about the status of patients at test-retest occasion or time intervals (the OSS, SST, PRTEE, and PRWHE) (23, 30, 31, 37). The quality assessment in four studies was rated as indeterminate since the Minimal Important Change (MIC) was not defined for those five PROs (the ASES, OSS, SST, FIHO, and PRWHE) (19, 23, 36, 37).

***Criterion validity***

Criterion validity was not assessed in any of the studies due to the lack of a gold standard in the evaluation of hand and upper extremity disability (41). 

***Hypothesis testing for construct validity***

Construct validity was assessed in 20 (90%) studies for all PROs. The most common comparator instruments were the DASH (14 studies) and SF-36 (11 studies). The range of correlation coefficient of the PROs with the comparators for the construct validity was 0.12 to 0.84. The risk of bias assessment in 8 studies was rated as very good as the authors reported the mean and standard deviation of the scores and used a valid instrument as a comparator (the OSS, OSIS, SST, and WORC) (23–26, 30, 34–36). The quality assessment in 11 studies was rated as insufficient since the results were not in accordance with the priori set hypothesis (21–23, 25–27, 29, 36, 39, 40). 

***Responsiveness***

Three studies (13%) assessed responsiveness and reported the Area Under Curve (AUC) of the four PROs (The DASH, SPADI, SAS, and PREE); therefore, the risk of bias was rated as very good for them (20, 27, 29). The value of AUC for three PROs was more than 0.7 (The DASH, SPADI, and PREE), and quality assessment was rated as sufficient (20, 29). The AUC value for the SAS was 0.67 and the quality assessment was rated as insufficient (27). None of the studies reported Standard Response Mean (SRM) or effect size as indexes of responsiveness. 

***Floor and ceiling effects***

If more than 15% of respondents of a PRO get the lowest and highest possible score, floor and ceiling effects are present. In 5 (22%) studies (6 PROs) floor and ceiling effects were assessed, and all of them found no floor and ceiling effects (The DASH, ASES, OSS, SST, WORC, and SAS) (19, 23, 26, 27, 39).

## Discussion

This review synthesized the literature regarding the extent and methodological quality of translation, cross-cultural adaptation, and psychometric properties of the hand and upper extremity disability PROs in the Persian language. The results of this study concluded that evidence in terms of translation, cross-cultural adaptation, reliability, internal consistency, and construct validity is available for all Persian validated disability PROs. However, there is a lack of evidence on the structural validity, measurement error, cross-cultural validity, and responsiveness for most of them. 

Over 140 hand and upper extremity disability PROs are available in the literature, out of which, 17 PROs are available in the Persian language (9). The most used PROs that are common in the high-quality hand and upper extremity orthopedic research, are translated and adapted to the Persian language. The majority of the Persian validated disability PROs (47%) are region-specific, and the others are condition-specific (29%) and multi-region (24%). 

Selecting the best PRO to target the construct of interest in the desired population is essential in clinical research and mostly depends on the measurement and clinometric properties of that PRO (42, 43).

The guidelines for translation and cross-cultural adaptation developed by Beaton et al. was used in 75% of the studies (10). Most of the studies (67%) followed 80 to 100% of the recommended steps for cross-cultural adaptation and translation of a PRO. Forward translation, synthesis, expert committee review, and pilot testing were performed in accordance with the guidelines in most of the studies (75-100%). However, the quality of backward translation in most of the studies (52%) was not in accordance with the recommended guidelines for the positive rating.

None of the studies evaluated all psychometric properties, but internal consistency, test-retest reliability, and construct validity were evaluated for all the Persian disability PROs. 

The value of Cronbach’s alpha is available for all Persian validated disability PROs. Internal consistency is meaningful when it is presented with factor analysis as the interpretation of Cronbach’s alpha depends on the unidimensionality of a scale or subscales (16). It is recommended to ignore the value of Cronbach’s alpha on a total score in case that a scale is not unidimensional (17, 44). However, a high Cronbach’s alpha neither is a guarantee for the measurement of the construct of interest nor to report that the important concepts are missing (45, 46).

The test-retest reliability value in most of the validated PROs was high, except for SST and symptom severity scale of the BCTQ (25, 35). Low value of ICC (0.54) in the symptom severity scale of BCTQ was due to lack of a clear understanding of the translated items for patients. 

The wide variation in the range of ICC (0.31 to 0.78) reported for the SST could be due to the one-week time interval between the test-retest (25). However, the authors did not provide any evidence regarding the patients’ condition at the time intervals. In the assessment of test-retest reliability, time interval should be appropriate to make sure that patients are stable and to avoid recall bias (47).

To detect a change in a patient’s score that is due to systematic or random error, not due to true change, it is important to know the values of measurement error of a PRO (48). The values of measurement error were reported in most of the studies (53%) for the DASH, ASES, SPADI, OSS, SST, SAS, PRTEE, FIHO, and PRWHE (19, 20, 23, 27, 30-32, 36, 37). Lack of data on the value of SEM, MDC, or MCID in the other validated PROs, limits their interpretability in clinical settings. Lack of interpretability limits their efficiency in clinical practice to know the effect of treatment (49). 

Construct validity with defining a priori hypothesis on the expected direction (positive or negative) and magnitude (absolute or relative) of correlation is important in interpreting the results (44). Most of the studies (69%) showed low to moderate correlation with the comparator instruments. This could stem from choosing an inappropriate comparator instrument, for example, using VAS or NRS when a PRO is not measuring pain or lacking adequate psychometric properties of the comparator PRO in a population of interest (48).

Responsiveness is one of the important psychometric properties for each PRO to detect changes in a construct of interest over time (validity of a change score) (44). Considering that disability could be persistent over a long period, it is important to be measured properly (4, 9). Therefore, it is essential to be confident of the ability of a PRO that is being used to detect meaningful changes over time (50). The responsiveness of the DASH, SPADI, and SAS in shoulder problems, and the PREE in patients with elbow pathologies are available (20, 27, 29). The other 14 Persian disability PROs have no evidence of responsiveness in a specific population, that could be evaluated in future studies. 

None of the studies evaluated cross-cultural validity or measurement invariance. When adapting a PRO to a different culture or population, it is important to assess the cross-cultural validity of that PRO in the new context (age, gender, diagnosis, or any other relevant subgroup) to be a sufficient reflection of the original version (48). 

The ASES as a multiregional PRO for the assessment of pain and disability in the elbow and shoulder region, is only available for athletes with shoulder problems in the Persian population (9, 19, 51, 52). It is needed to assess the psychometric properties of the ASES in the general population with shoulder and elbow conditions.

As the results of PROs reflect patients’ health condition directly from individual’s perspectives, they are useful in leading clinical decision making. Clinicians need to choose the best available PRO that is reliable, valid, and responsive enough to evaluate the construct of interest in routine clinical practice. Our review appraised and provided a comprehensive overview of the available Persian PROs evaluating disability in the hand and upper extremity. The results of this review help clinicians to know the methodological quality of the available Persian hand and upper extremity disability PROs and highlight the gap in the evidence on this area. 

Overall, a reasonable number of PROs for the assessment of hand and upper extremity disability are available in the Persian language. The majority of them have sufficient and adequate evidence of reliability and validity to support their use in the target population. However, the evidence on the responsiveness property is lacking for most of the Persian disability PROs, limiting clinical utility of them in the routine practice. Further studies are needed to improve the quality of evidence in the areas that are still lacking.

**Table 1:** Patients’ characteristics and summary of the Persian validated hand and upper extremity disability PROs

**Patient Reported Outcome Measure**		**Year**	**Sample size**	**Male/** **Female**	**Age** **Mean (SD)**	**Population**
Multi Region (n=423)						
DASH (39)	Disabilities of the Arm, Shoulder and Hand	2008	221	133/88	45 (18)	Upper extremity disorders
Q-DASH (40)	Quick-Disabilities of the Arm, Shoulder and Hand	2015	202	73/129	41 (14)	Upper extremity conditions
Shoulder (n=1508)						
DASH (20)	Disabilities of the Arm, Shoulder and Hand	2015	200	100/100	39 (13)	Patients with shoulder disorders
ASES (19)	American Shoulder and Elbow Surgeons	2013	100	73/27	26 (6)	Athletes with shoulder problems
SPADI (21)	Shoulder Pain and Disability Index	2014	190	97/93	41 (15)	Shoulder problems
SPADI (20)	Shoulder Pain and Disability Index	2015	200	100/100	39 (13)	Patients with shoulder disorders
OSS (22)	Oxford Shoulder Score	2014	100	49/51	43 (15)	Degenerative or inflammatory shoulder problem
OSS (23)	Oxford Shoulder Score	2015	100	27/73	47 (13)	Shoulder disorders
OSIS (24)	Oxford Shoulder Instability	2016	150	-	28 (8)	Shoulder instability
SST (25)	Simple Shoulder Test	2016	148	78/70	48 (15)	shoulder conditions
SST (23)	Simple Shoulder Test	2015	100	27/73	47 (13)	Shoulder disorders
WORC (26)	Western Ontario Rotator Cuff Index	2008	120	-	47 (15)	Rotator cuff disorders
SAS (27)	Shoulder Activity Scale	2015	100	45/54	47 (14)	Patients with shoulder pain
Elbow (n=425)						
OES (28)	Oxford Elbow Score	2014	92	40/52	40 (15)	Elbow conditions
PREE (29)	Patient-Rated Elbow Evaluation	2017	73	43/30	41 (18)	Elbow pathologies
PRTEE (3^0)^	Patient-Rated Tennis Elbow Evaluation	2019	102	64/38	28 (8)	Tennis player
PRTEE (31^)^	Patient-Rated Tennis Elbow Evaluation	2020	68	24/44	-	Chronic lateral elbow tendinopathy
PRTEE (32)	Patient-Rated Tennis Elbow Evaluation	2020	90	34/56	46 (11)	Patients with tennis elbow
Hand and Wrist (n=793)						
PRWE (33^)^	Patient-Rated Wrist Evaluation	2016	20	10/10	-	CTS, DRF, and Scaphoid fracture
PRWE (34)	Patient-Rated Wrist Evaluation	2017	131	71/17	34 (14)	Patients with upper extremity conditions
BCTQ (35)	Boston Carpal Tunnel Syndrome Questionnaire	2018	142	19/123	-	Patients suffering from CTS
FIHO (36)	Functional Index of Hand Osteoarthritis	2017	72	45/27	56 (9)	Patients with hand osteoarthritis
PRWHE (37)	Patient-Rated Wrist/Hand Evaluation	2018	205	68/137	40 (15)	Patients with upper extremity conditions
MHQ (38)	Michigan Hand Questionnaire	2015	223	114/109	35 (15)	Common hand disorders

CTS: Carpal Tunnel Syndrome; DRF: Distal Radius Fracture.

**Table 2:** The value of each psychometric property for the Persian validated hand and upper extremity disability PROs

**Instrument**	**Construct validity**	**Cronbach’s alpha**	**ICC**	**SEM, MDC, MCID**	**AUC**	**Floor/ceiling effect**
DASH ^1^	SF-36: (-0.25 to -0.72) PF subscale: -0.65 VAS: 0.52	0.96	0.82 (0.64 to 0.92)	-	-	No
DASH ^3^	-	-	-	MCID: 25.4	0.77	-
Q-DASH ^2^	SF-36: (-0.24 to -0.56) PF subscale: -0.32 MHQ: -0.67	0.90	0.89 (0.81 to 0.92)	-	-	-
ASES ^4^	SF-36: (0.22 to 0.62) PF subscale: 0.54 DASH: -0.78	Total: 0.91	0.91	SEM: 6.14	-	No
SPADI ^5^	SF-36: (-0.46 to 0.31) PF subscale: -0.33 DASH: 0.61	Pain: 0.85 Disability: 0.94 Total: 0.94	Pain: 0.78 Disability: 0.84 Total: 0.84	-	-	-
SPADI ^3^	-	-	-	MCID: Total: 14.9 Pain: 26.4 Disability:23.9	Total: 0.82 Pain: 0.80 Disability: 0.80	
OSS ^6^	SF-36: (0.12 to 0.63) PF subscale: 0.48 DASH: -0.59	0.93	0.93 (0.90 to 0.96)	-	-	-
OSS ^7^	SST: 0.68	0.91	0.90 (0.77 to 0.95)	SEM = 6.8 (CI: ±13.3) SDC = 18.8.	-	No
OSIS ^8^	DASH:0.84 VAS: 0.79	0.90	0.94	-	-	-
SST ^9^	OSS: 0.58 DASH: -0.59 SF-36 (0.19 to 0.45) PF subscale: 0.45	0.84	0.61	-	-	-
SST ^7^	OSS: 0.68	0.73	0.94 (0.86 to 0.97)	SEM = 0.7 (CI: ±1.3) SDC = 3.7	-	No
WORC ^10^	SF-36 (0.42 to 0.69) PF subscale: 0.69 DASH: -0.78 VAS: -0.62	Physical symptoms: 0.88 Sports/recreation: 0.92 Work: 0.91 Lifestyle: 0.90 Emotions: 0.85 Total: 0.92	Physical symptoms: 0.94 Sports/recreation: 0.89 Work: 0.88 Lifestyle: 0.93 Emotions: 0.91 Total: 0.90	-	-	No
SAS ^1^1	SF-36: PF subscale: 0.21 SPADI disability: -0.13 SPADI total: -0.09	0.64	0.98	MCID: 1.5	0.67	No
OES ^12^	DASH: 0.80 SF-36: (-0.21 to -0.80) PF subscale: -0.58	Function: 0.95 Pain: 0.86 Social-psychological: 0.85 Total:0.92	Function: 0.9 Pain: 0.76 Social-psychological: 0.75 Total:0.85	-	-	-
PREE ^13^	DASH: 0.66 SF36 (-0.25 to -0.59) PF subscale: -0.25	Pain: 0.93 Function:0.95 Total: 0.91	Pain: 0.95 Function:0.97 Total: 0.98	-	0.97	-
PRTEE ^14^	DASH: Total: 0.88 Pain: 0.84 Function: 0.87	Total: 0.96 Pain: 0.92 Function: 0.96	Total: 0.95 Pain: 0.93 Function: 0.94	SEM: Total: 4.20 Pain: 2.71 Function: 2.27	-	-
PRTEE ^15^	DASH: 0.80	0.98	0.99	SEM: 0.21	-	-
PRTEE^ 16^	DASH: Total: 0.85 Pain: 0.74 Function: 0.91 VAS: Pain: 0.54	0.94	0.98	SEM: 5.4 MDC: 14.24	-	-
BCTQ ^1^9	Q-DASH: SSS: 0.64 FSS: 0.70	SSS: 0.86 FSS: 0.88	SSS: 0.54 FSS: 0.77	-	-	-
FIHO ^20^	NRS: 0.40 SF36: PCS: -0.57	0.89	0.89	SEM: 2 SDC: 5.4	-	-
PRWE ^17^	-	-	-	-	-	-
PRWE ^18^	DASH: 0.84 VAS: 0.54	0.93	0.95	-	-	-
PRWHE ^21^	DASH: 0.80	0.92	0.95 (0.82-0.97)	SEM: 4.5 MDC: 12.5	-	-
MHQ ^22^	DASH: -0.74 VAS: -0.19	HF: 0.65 ADL: 0.96 Normal work: 0.92 Pain: 0.79 Appearance: 0.83 Satisfaction: 0.83 Total:0.79	HF: 0.81 ADL: 0.78 Normal work: 0.86 Pain: 0.78 Appearance: 0.84 Satisfaction: 0.73 Total:0.84	-	-	-

AUC: Area Under the Curve; ICC: Intraclass Correlation Coefficient; SDC: Smallest detectable change; MDC: Minimal detectable change; SEM: Standard error of measurement; MCID: Minimal detectable change; PF: Physical functioning scale; PCS: Physical component scale; SSS; Symptom severity scale; FSS: Function severity scale; NRS: Numeric rating scale; VAS: Visual analogue scale; HF: Hand Function subscale; ADL: Activities of Daily Living;

**Table 3:** Cross-cultural adaptations steps of the Persian validated hand and upper extremity disability PROs

**PRO**	**Translation**	**Synthesis**	**Back translation**	**Expert committee review**	**Pretesting**	**% of positive rating**
DASH (39)	+	+	+	+	+	100%
ASES (19)	+	+	-	+	0	60%
SPADI (21)	+	+	-	+	+	80%
OSS (22)	+	+	-	+	0	60%
OSS (23)	+	+	+	+	+	100%
OSIS(24)	+	+	+	+	+	100%
SST (25)	+	+	-	?	+	60%
SST (23)	+	+	+	+	+	100%
WORC (26)	+	+	+	+	+	100%
SAS (27)	+	+	-	0	+	60%
OES (28)	+	+	-	+	+	80%
PREE (29)	+	+	+	+	+	100%
PRTEE (30)	+	+	-	+	0	60%
PRTEE (31)	+	?	+	+	+	80%
PRTEE (32)	+	+	+	+	+	100%
PRWE (33)	+	+	+	+	+	100%
PRWE (34)	+	?	-	+	+	60%
BCTQ (35)	+	+	?	0	+	60%
FIHO (36)	+	+	+	?	+	80%
PRWHE (37)	+	+	-	+	+	80%
MHQ (38)	+	+	-	+	+	80%

+ = Positive rating; − = negative rating; 0 = no information available; ? = unclear.

**Table 4:** Risk of bias and quality assessment of the Persian validated disability PROs based on COSMIN risk of bias checklist and updated criteria for good measurement properties

**Instrument**	**Structural validity**	**Internal consistency**	**Reliability**	**Measurement error**	**Construct Validity**	**Responsiveness**
DASH (39)	Adequate (?)	Very good (+)	Adequate (+)		Adequate (-,-)	
DASH (20)						Very good (+)
Q-DASH (40)		Very good (+)	Adequate (+)		Adequate (-,-)	
ASES (19)		Inadequate (+)	Doubtful (+)	Doubtful (?)	Adequate (-,+)	
SPADI (21)		Very good (+)	Doubtful (+)		Adequate (-,-)	
SPADI (20)						Very good (+)
OSS (22)		Very good (+)	Adequate (+)		Adequate (-,-)	
OSS (23)	Adequate (?)	Very good (+)	Adequate (+)	Adequate (?)	Very good (-)	
OSIS (24)		Very good (+)	Adequate (+)		Very good (+,+)	
SST (25)		Very good (+)	Adequate (-)		Very good (-,-,-)	
SST (23)	Adequate (?)	Very good (+)	Adequate (+)	Adequate (?)	Very good (-)	
WORC (26)		Very good (+)	Adequate (+)		Very good (+,-,-)	
SAS (27)	Very good (+)	Very good (-)	Very good (+)		Adequate (-,-,-)	Very good (-)
OES (28)		Very good (+)	Adequate (+)		Adequate (+,-)	
PREE (29)		Very good (+)	Adequate (+)		Adequate (-,-)	Very good (+)
PRTEE (30)		Doubtful (?)	Adequate (+)	Adequate (-)	Very good (+)	
PRTEE (31)		Doubtful (?)	Adequate (+)	Adequate (+)	Adequate (+)	
PRTEE (32)		Doubtful (?)	Adequate (+)	Inadequate (-)	Adequate (+)	
PRWE (33)						
PRWE (34)		Inadequate (?)	Adequate (+)		Very good (+,-)	
BCTQ (35)		Very good (+)	Adequate (-,+)		Very good (+.-)	
FIHO (36)		Very good (+)	Very good (+)	Very good (?)	Very good (-,-)	
PRWHE (37)		Inadequate (?)	Adequate (+)	Adequate (?)	Adequate (+)	
MHQ (38)	Adequate (?)	Very good (+)	Adequate (+)		Adequate (+,-)	

+: Sufficient; -: Insufficient; ?: Indeterminate

## Data Availability

The data of the current study are available in detail, and it can be asked from the corresponding author whenever is needed.
